# Results from the Workshop “Problem Formulation for the Use of Gene Drive in Mosquitoes”

**DOI:** 10.4269/ajtmh.16-0726

**Published:** 2017-03-08

**Authors:** Andrew Roberts, Paulo Paes de Andrade, Fredros Okumu, Hector Quemada, Moussa Savadogo, Jerome Amir Singh, Stephanie James

**Affiliations:** 1 Center for Environmental Risk Assessment, International Life Sciences Institute Research Foundation, Washington, District of Columbia.; 2 Department of Genetics, Federal University of Pernambuco, Recife, Brazil.; 3 Ifakara Health Institute, Environmental Health and Ecological Sciences Thematic Group, Dar es Salaam, Tanzania.; 4 Institute for International Crop Improvement, Donald Danforth Plant Science Center, Saint Louis, Missouri.; 5 African Biosafety Network of Expertise, NEPAD Agency, Ouagadougou Node, University of Ouagadougou, Burkina Faso.; 6 Centre for the AIDS Programme of Research in South Africa, University of KwaZulu-Natal, Durban, South Africa.; 7 Dalla Lana School of Public Health, University of Toronto, Ontario, Canada.; 8 Foundation for the National Institutes of Health, Bethesda, Maryland.

## Abstract

Reducing the incidence of malaria has been a public health priority for nearly a century. New technologies and associated vector control strategies play an important role in the prospect of sustained reductions. The development of the CRISPR/Cas9 gene editing system has generated new possibilities for the use of gene-drive constructs to reduce or alter vector populations to reduce malaria incidence. However, before these technologies can be developed and exploited, it will be necessary to understand and assess the likelihood of any potential harms to humans or the environment. To begin this process, the Foundation for the National Institutes of Health and the International Life Sciences Institute Research Foundation organized an expert workshop to consider the potential risks related to the use of gene drives in *Anopheles gambiae* for malaria control in Africa. The resulting discussion yielded a series of consensus points that are reported here.

## Introduction

The control of malaria has been a global public health priority for almost 100 years.^1^ Concerted efforts in the 21st century by national governments, international bodies, and civil society organizations supporting public health programs to reduce the spread and impact of malaria have reduced the incidence of infection by 37% globally and mortality by 60% since 2000. In Africa, it is estimated that measures against malaria vectoring mosquitoes, notably the use of long-lasting insecticidal bed nets (LLINs) and house spraying with residual insecticides (IRS), have contributed ∼78% of all gains accrued against malaria since 2000. Nevertheless, more than 3 billion people remain at risk for malaria infection, and more than 200 million cases and over 400,000 deaths have been attributed to malaria in 2015.† This burden falls disproportionately on sub-Saharan Africa, where the deadliest malaria parasite, *Plasmodium falciparum*, coexists with the most efficient malaria vectors, including *Anopheles gambiae* and *Anopheles funestus.* †http://www.who.int/malaria/en/

Despite the key successes already achieved by major malaria vector control tools like LLINs and IRS, new complementary technologies are constantly being developed and evaluated for use in control programs to bridge existing gaps and accelerate progress toward eventual malaria elimination. Practical advances in molecular biology, particularly the successful use of the CRISPR/Cas9 gene editing machinery to create gene drive,‡ are envisioned to make more practical and realizable large-scale campaigns to drastically suppress malaria vector populations or alter these populations so they no longer transmit disease.^2^,^3^ However, before these methods can be developed and deployed, it will be necessary to assess their potential to cause harm to human health and the environment. ‡“Gene drive” here refers to the altered inheritance of any allele such that it is transmitted to most offspring, rather than the 50% expected through Mendelian inheritance. This can be achieved, theoretically, through a variety of mechanisms including, for example, naturally occurring selfish genetic elements.

With the goal to inform research programs, public health and donor organizations and government regulators about plausible risks related to potential uses of gene-drive technology in mosquitoes, the Foundation for the National Institutes of Health convened a 3-day workshop involving expert participants with diverse perspectives to identify the hazards. The workshop, which was facilitated by the International Life Sciences Institute Research Foundation's Center for Environmental Risk Assessment, applied a problem formulation approach to identify plausible risks using case studies. The case studies were developed to illustrate realistic applications of gene drive for malaria vector control in sub-Saharan Africa, particularly focusing on *A. gambiae*.

## Conduct of the Workshop

International experts including researchers, public health officials, and regulatory officials participated in the 3-day program. The first day consisted of background lectures on 1) the use of CRISPR/Cas-9 system to drive genetic constructs,^4^,^5^ 2) the biology of *A. gambiae*,^6^ and 3) the problem formulation process for planning risk assessment and identifying relevant concerns.^7^–^9^ Participants then conducted a problem formulation exercise based on case studies providing background information and hypothetical examples illustrating potential uses of gene-drive strategies for population suppression and population alteration by either introducing genes encoding novel proteins, or editing endogenous genes in the mosquito. The end result was the identification of pertinent environmental/ecological protection goals that could plausibly be impacted by releasing genetically modified mosquitoes, followed by discussion of how those protection goals might reasonably be impacted. Consensus from these discussions was then drafted to inform ongoing dialogue around use of gene-drive strategies against malaria vectors, and to guide future in-depth risk assessments on individual specific technologies employing population suppression or population alteration. Although the many rich discussions could not be captured in a series of consensus statements, and nothing in these points is intended to preclude consideration of other issues, we believe the stated consensus notes will inform research and product development efforts, specifically with regard to priorities for data collection and analysis for environmental and ecological risk assessments.

## Consensus Points

Two types of uses for gene drives in mosquitoes were considered. Both are intended to contribute to the control of malaria, but have different implications for potential environmental interactions.

- Gene-drive mosquitoes for population suppression are designed to eventually reduce in numbers in the environment over a relevant time.
- Gene-drive mosquitoes for population alteration are designed to persist in the environment over a relevant time.

In light of considerations for both of these applications of the technology, participants discussed broad areas of environmental protection that would be pertinent to consider when performing environmental risk assessment for the use of gene drive in *A. gambiae*. The first consensus was thus on which broad protection goals were pertinent, and which ones were nonpertinent (Box 1

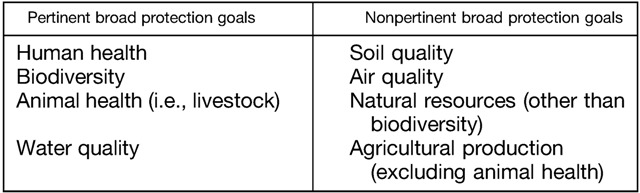

).

Participants acknowledged that *A. gambiae* does not interact substantially with the air and soil in ways that are important for environmental quality, or have relevant interactions with natural resources and agricultural production other than feeding on livestock. Instead, the relevant interactions in the environment were related to human health and pathogen transmission, interactions of *A. gambiae* with other organisms in the environment, and its potential to transmit livestock pathogens. Because the lifecycle of *A. gambiae* involves an aquatic stage, and this stage feeds on microorganisms, there was consensus that water quality should be considered, though these interactions were viewed as minor since the mosquitoes prefer small and transient water bodies.

## Consensus Points Related to Pertinent Protection Goals

### Human health.

- The relevant interaction for human health is biting.
- Incidental exposure through inhalation, ingestion, etc. is not likely to result in any significant levels of exposure leading to harm to human health.
- Proteins introduced into *A. gambiae*, including components of the gene drive and markers, should be considered with respect to toxicity and allergenicity potential.
- Horizontal gene flow to humans is extremely unlikely to occur.
- Because *A. gambiae* is an important disease vector, consideration should be given to potential alterations in disease transmission.
- This includes altered *P. falciparum* transmission or virulence, other human malarial transmission as well as altered transmission of other diseases.

Female *Anopheles* mosquitoes interact with humans through biting to obtain blood nutrients necessary for egg production. Although incidental exposures are possible through inhalation and ingestion of the introduced gene-drive mosquitoes, these exposures were considered unlikely to cause harm. However, *A. gambiae* is known to transmit other pathogens in addition to *P. falciparum*. Participants identified potential alterations to disease transmission as an important area of consideration for any future application, understanding that the ability to affect disease transmission would depend on the specific mechanism for achieving the desired phenotype. Changes in endogenous proteins or gene functions in *A. gambiae* should be assessed for their ability to alter rates of transmission or virulence of *P. falciparum* as well as alter the transmission of other *Anopheles*-transmitted diseases. Introduced proteins should be considered for potential toxicity and allergenicity to humans if the proteins are likely to come in contact with humans through biting(e.g., to be present in saliva of the transgenic mosquito). Participants did not think horizontal gene flow was likely to lead to harm to humans because of the extremely rare frequency of occurrence^10^–^12^ and the expectation that introduced genes would not be present in mosquito saliva.

### Biodiversity.

- *Anopheles gambiae* is not a “keystone” species in the environment and is not known to provide any nonredundant ecosystem services.
- Changes in population size or even elimination of *A. gambiae* from a particular environment are unlikely to harm biodiversity or ecosystem services. This is based on existing knowledge and experience with vector control programs.
- *Anopheles gambiae* interacts with other species by feeding on them, being consumed as prey, or competing with them.
- These interactions may require consideration for species of relevance to the assessment such as threatened, endangered, or valued species.
- Incidental contact between organisms and *A. gambiae* carrying gene drives is not likely to lead to harms to those organisms, compared with interactions with other *A. gambiae*.
- *Anopheles gambiae* is not known to be the sole or primary food source for any organism, with the possible exception of a few species of spider known to prefer anophelines.
- Removing *A. gambiae* from the environment is unlikely to harm species that feed on it, due to the availability of other prey, including anophelines.
- Consideration should be given to any proteins introduced into *A. gambiae* (including gene-drive components or markers) for toxicity to other species.
- Gene flow to other species within the *A. gambiae* s.l. complex through hybridization is likely, and does not create additional pathways to harm.
- Horizontal gene transfer is not likely to occur to other organisms on any relevant time scale and is not a pertinent pathway to harm.

Biodiversity as a protected endpoint is complex, and any risk assessment is dependent on identification of what aspects of biodiversity are considered valuable, and what changes in biodiversity are considered to be harmful or undesirable. Presentations and participants from sub-Saharan countries provided some context for how biodiversity is considered in the region, and participants also used experience with existing vector control programs to inform their discussions. It was generally agreed that populations of *A. gambiae* are not locally valued or protected. Rather they are treated as a nuisance and a public health threat and subject to a variety of programs to reduce populations. Most participants did not consider reduction or alteration of these populations, in and of themselves, to represent a harm to biodiversity.

The potential for ecological harm as a result of changes or reductions in population is normally assessed through consideration of interactions with other organisms in the environment, and *A. gambiae* primarily interacts with other organisms through feeding on them (humans and other large mammals), serving as prey, or as a competitor with other mosquito species in aquatic habitats during larval stages. As mentioned above for human health, toxicity of any introduced proteins for other species should be considered. Drawing from presented materials, and information contained in the case studies, participants concluded that the loss of *A. gambiae* from a particular environment would be unlikely to cause ecological harm. Although this species may be preyed on by many animals, it does not constitute a significant or crucial portion of the diet for any known species. Participants noted reports that certain spider species prefer to feed on anophelines when available, but the presence of other food sources in any particular environment makes the contribution of changes to *A. gambiae* populations less likely to lead to harm.^13^

It was also noted that gene flow to other members of what has been known as the *A. gambiae* complex (*A. gambiae* s.l.), historically consisting of seven or eight morphologically indistinguishable species that have some potential for hybridization, is likely to occur. However, the movement of gene drive to these species would be expected to produce similar impacts on the environment as in *A. gambiae*. Participants recognized that this gene flow should be accounted for in risk assessment, but the consensus was that this would not produce any additional pathways to harm for other valued species or assessment endpoints. Indeed, because several of these species also transmit malaria, acquisition of the gene-drive construct could well be beneficial in further reducing disease transmission.

### Animal health (livestock).

- Potential harm could result from altered pathogen transmission dynamics to livestock.
- Harm resulting from other mechanisms, including toxicity from introduced proteins, was considered unlikely.

Similar to humans, livestock animals interact with *A. gambiae* primarily as targets of feeding. Therefore, the consensus among participants was that potential for altered disease transmission to livestock would need to be addressed in risk assessments. Again, similar to humans, livestock may ingest or inhale mosquitoes incidentally, but these exposures are expected to be low and participants did not think harm from these exposures would be consequential when considering the use of gene drives for population alteration or suppression in *A. gambiae*. Although the possibility of toxicity of proteins to livestock was considered remote, this concern would be dealt with through consideration of toxicity to humans, as noted above.

### Additional considerations.

The primary purpose of the workshop was to consider the potential for environmental or ecological harm from the use of gene-drive technology in *A. gambiae*. This included consideration for human health as a component of the environment. However, through the course of the exercise, several recurring discussions suggested that, although not directly related to environmental risk assessment, some basic consensus points on the use of this particular technology could be easily agreed and should be considered by product developers, governments, and public health programs.

- The use of gene drives in *A. gambiae* should be considered as a complementary strategy to other vector control methods and malaria mitigation strategies.
- The potential harms identified for the use of gene drive in *A. gambiae* should be considered in the context of other vector control methods and malaria mitigation strategies.
- Failure to sustain a successful malaria vector control strategy can have harmful effects on malaria incidence.
- This is not unique to gene drive, and would be the same for other malaria control or eradication techniques.
- The ability to control disease resurgence needs to be sustained and availability of effective additional control methods assured.

The crux of these conclusions is to the importance of placing the use of gene drives in the context of existing malaria mitigation strategies. Gene-drive technology offers opportunities for well-organized large-scale vector control operations with potential to drastically suppress or alter malaria vector populations to reduce or stop transmission. Decades of experience with vector control and malaria control has provided valuable insight on the need for maintaining the viability of multiple control methods. Despite the novelty of the molecular biology and the excitement surrounding the technology, there was strong consensus that the idea that a single technology, or a single application of a technology, would be sufficient to eradicate malaria transmission is unrealistic. Gene-drive strategies hold promise as a strong complement to other methods, but they are not intended and should not be considered to be a single solution to malaria mitigation. The use of gene drives, and their environmental risk assessment, will need to be considered in this context, and be informed by relevant experiences with other control methods.

## Conclusion

Problem formulation is a process for incorporating legal, social, and scientific context into the planning for an environmental or ecological risk assessment. It is a useful exercise for formally identifying the types of information that will be useful in conducting an assessment. This workshop provided an opportunity for experts to consider, through a case study approach, the ways a gene-drive strategy in *A. gambiae* for mitigating malaria might harm the environment or human health. Definitions of harm were derived from participants' understanding of environmental protection goals in sub-Saharan Africa and informed by presentations at the workshop as well as prior experience. This was a practical necessity, and should not be construed to minimize the importance or relevance of social or ethical discussions which will be an important component of decision-making around the use of gene drive, as they would be with any important decision or policy that deals with the environment and human health.

Although the discussions were wide ranging, there was a great deal of commonality in the resulting analyses. Human health considerations related to disease transmission were considered important for analysis in the context of the risk assessment. Harms to livestock health were considered unlikely, but the potential for altered pathogen transmission was identified as a relevant endpoint for assessment. Although harmful impacts to biodiversity were generally considered unlikely due to suppression or alteration of *A. gambiae* populations, there was consensus on the important ecological interactions that should be considered in specific risk assessments.

It is also important to consider that there may be different perspectives on environmental protection goals, moral or ethical considerations for the use of the technology. This effort used existing environmental protection goals as a baseline for consideration and did not make any de novo attempt to address ethical or social implications from the deployment of gene-drive technology. The authors would encourage these considerations to be taken up in appropriate forums, and particularly in communities where the use of gene-drive technologies would likely occur. This article therefore also provides a basis to support continued engagement of affected communities, and other relevant stakeholders as gene-drive technologies continue to be developed.
